# Development, Validation, and Application of a Diabetes Drug Discontinuation Questionnaire: A Multicentric Observational Study

**DOI:** 10.7759/cureus.100966

**Published:** 2026-01-06

**Authors:** Vivek Agarwal, Mukulesh Gupta, Rajiv Awasthi, Kumar Prafull Chandra, Nitin R Gupta, Santoshkumar Chaubey, Arunkumar R Pande, Dinesh Kumar

**Affiliations:** 1 Endocrinology, RR Diabetes and Heart Care Centre, Lucknow, IND; 2 Diabetes and Endocrinology, Udyan Health Care, Lucknow, IND; 3 General Medicine, Prarthana Clinic & Diabetes Care Centre, Lucknow, IND; 4 General Medicine, Health City Vistaar Hospital, Lucknow, IND; 5 Endocrinology, Lucknow Hormone Center, Lucknow, IND; 6 General Medicine, Cairns and Hinterland Hospital and Health Service, Cairns, AUS; 7 Endocrinology, Lucknow Endocrine Diabetes and Thyroid Clinic (LEDTC), Lucknow, IND; 8 General Medicine, Harsha Clinic, Lucknow, IND

**Keywords:** diabetes, discontinuation, item response theory, medication adherence, questionnaire validation

## Abstract

Background: Medication adherence refers to patients taking their prescribed treatments at the correct dose, timing, and frequency. In contrast, medication discontinuation involves sustained interruption of the entire regimen, with greater risks of metabolic decompensation and complications. Despite its clinical significance, anti-diabetes medication discontinuation remains poorly understood, with no standardized definitions or validated assessment tools.

Objective: To develop and validate a culturally adapted, self-administered questionnaire to assess the prevalence, duration, and factors linked to discontinuation of anti-diabetes medication.

Methods: This multicenter observational study was conducted across 11 diabetes care centers in Northern India. A structured questionnaire was developed through expert consensus and patient pre-testing, followed by psychometric validation. The validated instrument was then administered to a large cohort of adults with diabetes to assess discontinuation patterns and associated factors.

Results: Among 747 participants (mean age: 55.98 years; 54.2% male), 189 individuals (25.3%) reported at least one episode of medication discontinuation lasting more than seven consecutive days. Discontinuation was frequently prolonged: 36.3% of affected participants discontinued therapy for one to six months, and 29.4% for more than one year. Compared with those who remained on therapy, individuals who discontinued had higher average glycemic levels, including a mean HbA1c approximately 0.5% higher, along with higher fasting and post-prandial glucose values. The most commonly reported reasons for discontinuation were medication unavailability (34/197; 17.3%), fear of hypoglycemia (33/197; 16.8%), perceived good glycemic control (29/197; 14.7%), and belief that medication was unnecessary (27/197; 13.7%). Discontinuation was more common among individuals with behavioral risk factors and microvascular complications, while concurrent use of cardiovascular medications was associated with greater treatment persistence. Most participants considered discontinuation unjustified and expressed strong support for digital reminder systems.

Conclusions: Medication discontinuation affects approximately one in four diabetes patients, often for extended periods. Unlike adherence, discontinuation is rarely measured. This validated questionnaire provides the first reliable tool to assess this behavior in India, highlighting knowledge gaps and access barriers as primary drivers, rather than socioeconomic factors. Interventions should focus on patient education and digital adherence support systems.

## Introduction

Type 2 diabetes mellitus (T2DM) remains a major global health challenge with complex, multifactorial determinants. According to the International Diabetes Federation (IDF) Diabetes Atlas, 11th edition (2025), an estimated 589 million adults aged 20-79 years are currently living with diabetes worldwide. This figure is projected to increase to 853 million by 2050, with more than 40% remaining undiagnosed and nearly 80% of the burden concentrated in low- and middle-income countries (LMICs) [1].

India contributes disproportionately to the global diabetes burden. The ICMR-INDIAB study (2023) reported a national prevalence of 11.4% for diabetes and 15.3% for prediabetes, corresponding to approximately 101 million adults living with T2DM [2]. The Global Burden of Disease (GBD) 2016 study further showed that the number of people with diabetes in India rose from 26 million in 1990 to 65 million in 2016, accompanied by a 39.6% increase in disability-adjusted life years (DALYs), the sharpest rise among major non-communicable diseases [3].

Effective management of diabetes depends on sustained pharmacotherapy. Medication adherence, defined as the extent to which patients take medicines at the prescribed dose, timing, and frequency, is fundamental to achieving and maintaining glycemic control. Suboptimal adherence is consistently linked to poor outcomes, including increased complications and higher healthcare costs [4]. According to a landmark World Health Organization (WHO) report, adherence to long-term therapies averages only about 50%, and in chronic conditions may reach no more than 75% [5].

Discontinuation is conceptually distinct from adherence. While non-adherence typically refers to irregular intake or missed doses, discontinuation denotes the complete cessation of all prescribed anti-diabetes medications for a sustained period, a more severe behavior with potentially greater clinical consequences [6]. In infectious disease programs, discontinuation is clearly defined and systematically monitored: tuberculosis control frameworks classify “lost to follow-up” as treatment interruption for ≥2 months [7], while HIV care programs define treatment gaps of ≥90 days [8]. In contrast, diabetes care lacks standardized definitions or validated tools to capture discontinuation, limiting comparability across studies and constraining the development of targeted interventions.

Most research on diabetes medication-taking behaviors has focused on adherence, typically assessed through self-report instruments such as the Diabetes Self-Management Questionnaire (DSMQ) and, historically, the Morisky scales [9-11]. While these tools provide valuable insights into overall compliance, they do not distinguish or quantify episodes of sustained treatment discontinuation. As a result, the magnitude, duration, and determinants of discontinuation remain poorly characterized, particularly within the Indian context.

In this study, anti-diabetes drug discontinuation was defined as the cessation of all prescribed oral or injectable medications for more than seven consecutive days. This threshold was chosen to exclude minor lapses due to forgetfulness, temporary unavailability of drugs, short-term travel, or transient social and religious practices, while capturing interruptions of clear clinical relevance.

The primary objective was to develop and validate a culturally adapted, self-administered questionnaire in Hindi and English to assess the prevalence, duration, and determinants of anti-diabetes medication discontinuation across multiple centers in Northern India.

## Materials and methods

Study design and setting

This multicenter, cross-sectional observational study was conducted between 2021 and 2023 across 11 tertiary and secondary care centers in Lucknow, India. The study protocol was prospectively registered with the Clinical Trials Registry of India (CTRI: REF/2020/07/035433) and received ethical approval from institutional review boards at all participating sites. The study followed a structured three-phase design (Table 1): (1) Phase I: Development of the Diabetes Drug Discontinuation (DDD) questionnaire; (2) Phase II: Validation of the instrument; and (3) Phase III: Application of the validated tool in a large patient cohort.

**Table 1:** Study schema

Study Phase	Details
Phase I: Questionnaire Development	Expert panel comprising endocrinologists, diabetologists, and behavioral scientists Initial pool of 20 items refined to a final set of 15 items Content validity confirmed by three independent experts Pre-testing conducted in 20 patients
Phase II: Validation	Conducted in 100 patients with a history of medication discontinuation Psychometric evaluation using Item Response Theory and Rasch modeling Internal consistency assessed (Cronbach’s α = 0.88) Forward–back translation performed (Hindi ↔ English)
Phase III: Application	Administered to 747 patients across participating study centers Data collection included demographics, laboratory parameters, and comorbidities Statistical analyses included correlation tests, chi-square tests, and regression analyses Medication discontinuation defined as cessation for more than 7 consecutive days

Sample size calculation

Sample size was determined using population-based estimates. With Lucknow’s population of 3.56 million, an adult proportion of 58.9%, a diabetes prevalence of 8.8% (IDF estimates), and an expected discontinuation prevalence of 16.9% from prior studies, the minimum required sample size was calculated as 216 patients at 95% confidence and 5% margin of error. After applying a design effect of three to account for clustering and non-response, the adjusted minimum sample was 648. Ultimately, 747 patients were enrolled, exceeding the required sample size.

Participants

Eligible participants were adults (≥18 years) with a confirmed diagnosis of diabetes, receiving pharmacological therapy for at least one month in accordance with American Diabetes Association (ADA) guidelines, and willing to provide written informed consent. Patients were excluded if they were using alternative medicine exclusively, had experienced an acute illness requiring hospitalization within the preceding month, were unable to read or comprehend the questionnaire, or had cognitive impairment preventing reliable self-care (Table 2).

**Table 2:** Cohort flowchart

Study Component	Details
Participant Overview	(1) Patients screened: N = 1,024. (2) Excluded: N = 277, Unable to read/comprehend questionnaire (n = 102), Exclusive alternative medicine use (n = 61), Recent acute illness/hospitalization <1 month (n = 48), Cognitive impairment (n = 25), Declined consent (n = 41). (3) Eligible participants: N = 747. (4) Included in final analysis: N = 747
Inclusion Criteria	Age ≥18 years, Confirmed diagnosis of diabetes mellitus, On pharmacological anti-diabetes therapy for ≥1 month, Able to read and understand the questionnaire, Provided written informed consent
Exclusion Criteria	Exclusive use of alternative medicine, Acute illness requiring hospitalization in the preceding month, Cognitive impairment preventing reliable participation, Inability to read or comprehend the questionnaire, Refusal to provide informed consent

Three-phase methodology

Phase I: Questionnaire Development

A panel of 10 experts, including endocrinologists, diabetologists, and behavioral scientists, generated a draft pool of 20 items in Hindi addressing treatment discontinuation, perceptions of glycemic control, and reasons for cessation. After iterative review, 15 closed-ended items were finalized (Appendix 1, Supplementary Material). Three independent subject experts assessed content validity, and suggested revisions were incorporated through a consensus process.

Phase II: Validation

Pilot testing was conducted in 100 participants to assess clarity, cultural appropriateness, and item performance. Psychometric validation included Item Response Theory (IRT) to evaluate item discrimination and difficulty parameters, as well as Rasch modeling to examine unidimensionality, measurement precision, and differential item functioning across demographic groups. Internal consistency was measured using Cronbach’s alpha, with a target reliability coefficient ≥0.80 [12-15]. Forward-back translation ensured semantic equivalence between Hindi and English versions.

Phase III: Application

The validated DDD questionnaire was administered to 747 patients across the participating centers. Trained study personnel also collected demographic characteristics, clinical history, comorbidities, anthropometric measurements, and laboratory values (fasting plasma glucose, post-prandial plasma glucose, HbA1c, serum creatinine, estimated glomerular filtration rate) using standardized case record forms. Socioeconomic status was evaluated using the Kuppuswamy scale, updated with the February 2020 Consumer Price Index (328; base year 2001) [16].

Statistical analysis

Descriptive statistics were used to summarize demographic and clinical characteristics, as well as the responses to the questionnaire. Pearson correlation coefficients were calculated to assess associations between discontinuation and continuous variables. Chi-square tests were used for categorical variables. Multivariate logistic regression identified independent predictors of discontinuation, adjusting for potential confounders. All statistical tests were two-tailed, and significance was set at p<0.05. Analyses were performed using Statistical Product and Service Solutions (SPSS, version 26; IBM SPSS Statistics for Windows, Armonk, NY).

Disclaimer

All study participants were clinic-based, and the findings reported in the study may not be generalizable to community-based or untreated populations.

## Results

Phase I: Questionnaire development

A multidisciplinary panel of endocrinologists, diabetologists, and behavioral scientists generated an initial pool of 20 items addressing treatment discontinuation. Through iterative review and expert consensus, the tool was refined to 15 closed-ended questions covering discontinuation duration, perceptions of glycemic control, and reasons for cessation. Content validity was confirmed by three independent experts, and pre-testing in 20 patients demonstrated clarity, cultural appropriateness, and ease of comprehension.

Phase II: Questionnaire validation

Psychometric evaluation was conducted in 100 patients with a history of at least one discontinuation episode. Pilot testing confirmed feasibility and acceptability. Item Response Theory (IRT) analysis demonstrated appropriate item discrimination (0.60-1.40) and difficulty parameters. Rasch modeling confirmed unidimensionality (infit/outfit mean squares: 0.8-1.2) with person-separation reliability of 0.85. Internal consistency was excellent (Cronbach’s alpha: 0.88). Forward-back translation established semantic equivalence between the Hindi and English versions.

Phase III: Application of the questionnaire

Study Population

A total of 747 patients were enrolled (mean age: 55.98 ± 11.30 years; 54.2% male; 55.7% urban). The mean duration of diabetes was 2.95 ± 0.96 years. Baseline laboratory values were fasting plasma glucose 148.20 ± 61.99 mg/dL, post-prandial plasma glucose 209.80 ± 89.51 mg/dL, and HbA1c 8.08 ± 2.07% (Tables 3-4).

**Table 3:** Baseline demographic and clinical characteristics of the study population n = number of participants

Characteristic	Total Population (N = 747)
Gender	
Male, n (%)	405 (54.2)
Female, n (%)	342 (45.8)
Place of residence	
Urban, n (%)	416 (55.7)
Rural, n (%)	331 (44.3)
Behavioral risk factors	
Smoking, n (%)	37 (5.0)
Tobacco use (any form), n (%)	100 (13.4)
Alcohol consumption, n (%)	71 (9.5)
Family history of diabetes, n (%)	385 (51.5)
Education level	
Illiterate, n (%)	52 (7.0)
Less than middle school, n (%)	43 (5.8)
Middle school certificate, n (%)	76 (10.2)
High school certificate, n (%)	86 (11.5)
Secondary certificate, n (%)	108 (14.5)
Graduate degree, n (%)	235 (31.5)
Post-graduate degree, n (%)	147 (19.7)
Employment status	
Unemployed, n (%)	215 (28.8)
Unskilled worker, n (%)	18 (2.4)
Semi-skilled worker, n (%)	45 (6.0)
Skilled worker, n (%)	140 (18.7)
Semi-professional, n (%)	95 (12.7)
Professional, n (%)	185 (24.8)
Comorbidities	
Type 2 diabetes mellitus, n (%)	742 (99.3)
Hypothyroidism, n (%)	154 (20.6)
Hypertension, n (%)	427 (57.2)
Macrovascular complications, n (%)	75 (10.0)
Microvascular complications, n (%)	154 (20.6)
History of hypoglycemia, n (%)	159 (21.3)
Current medications	
Antihypertensive use, n (%)	408 (54.6)
Statin use, n (%)	371 (49.7)
Aspirin use, n (%)	221 (29.6)
Home glucometer availability, n (%)	542 (72.6)
Number of oral anti-diabetic drug pills per day	
1 pill, n (%)	113 (15.1)
2 pills, n (%)	261 (34.9)
3 pills, n (%)	184 (24.6)
≥4 pills, n (%)	189 (25.3)

**Table 4:** Baseline clinical and laboratory parameters of the study population Mean = arithmetic average; SD = standard deviation; mmHg = millimeters of mercury; bpm = beats per minute; mg/dL = milligrams per deciliter; mL/min/1.73 m² = milliliters per minute per 1.73 square meters

Parameter	Mean ± Standard Deviation
Age (years)	55.98 ± 11.30
Height (centimeters)	160.70 ± 9.45
Weight (kilograms)	69.63 ± 13.45
Systolic blood pressure (millimeters of mercury)	133.70 ± 18.51
Diastolic blood pressure (millimeters of mercury)	78.19 ± 11.02
Pulse rate (beats per minute)	89.25 ± 13.95
Duration of diabetes (years)	2.95 ± 0.96
Fasting plasma glucose (milligrams per deciliter)	148.20 ± 61.99
Post-prandial plasma glucose (milligrams per deciliter)	209.80 ± 89.51
Glycated hemoglobin (%)	8.08 ± 2.07
Serum creatinine (milligrams per deciliter)	1.01 ± 2.08
Estimated glomerular filtration rate (milliliters per minute per 1.73 square meters)	87.08 ± 23.43

Prevalence and Duration of Discontinuation

**Figure 1:**
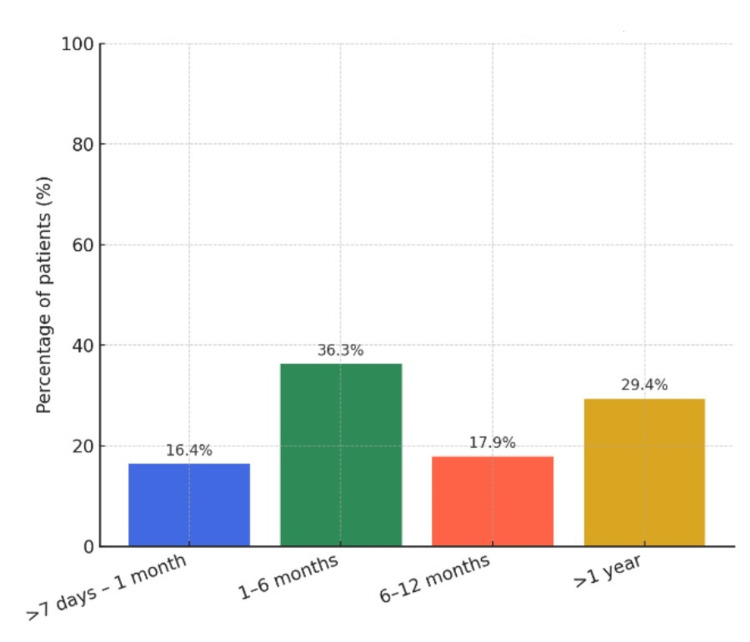
Duration of discontinuation

A total of 189 participants (25.3%) reported at least one episode of discontinuation lasting >7 days. Among 197 respondents who provided detailed histories, 33 (16.4%) discontinued for >7 days-1 month, 73 (36.3%) for 1-6 months, 36 (17.9%) for 6-12 months, and 59 (29.4%) for more than one year (Figure 1).

Factors Associated With Discontinuation

Compared with those who remained on therapy, patients who discontinued were younger (53.35 ± 11.79 vs. 56.87 ± 11.00 years; p = 0.0002) and had a shorter diabetes duration (2.83 ± 0.88 vs. 3.00 ± 0.98 years; p = 0.034). Glycemic indices were consistently worse in the discontinuation group: HbA1c 8.44 ± 2.23% vs. 7.95 ± 2.00% (p = 0.0047); fasting glucose 165.10 ± 77.91 vs. 142.30 ± 54.27 mg/dL (p = 0.0003); and post-prandial glucose 233.80 ± 111.20 vs. 201.30 ± 78.75 mg/dL (p = 0.0003).

**Figure 2:**
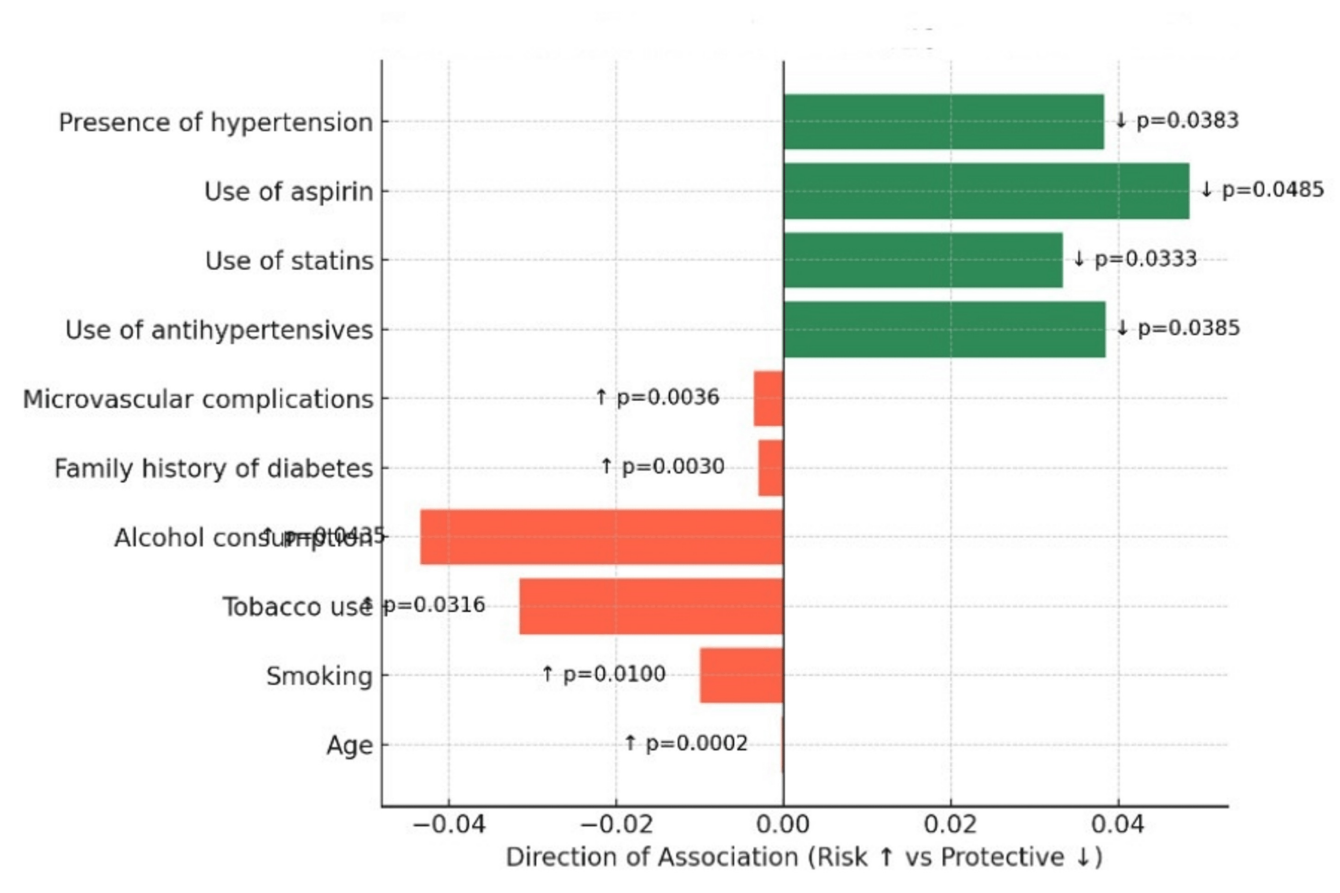
Factors associated with discontinuation

Correlation analyses identified positive associations with age (r = 0.1355; p = 0.0002), smoking (p = 0.01), tobacco use (p = 0.0316), alcohol consumption (p = 0.0435), family history of diabetes (p = 0.003), and microvascular complications (p = 0.0036). Protective associations included use of antihypertensives (p = 0.0385), statins (p = 0.0333), and aspirin (p = 0.0485), as well as the presence of hypertension (p = 0.0383). No significant associations were observed for gender, education, employment status, place of residence, BMI, renal function, or blood pressure (Figure 2). Although several correlations reached statistical significance, the observed effect sizes were small in magnitude (r values approximately 0.10-0.15), indicating weak linear associations between individual factors and medication discontinuation.

Reasons for Discontinuation

**Figure 3:**
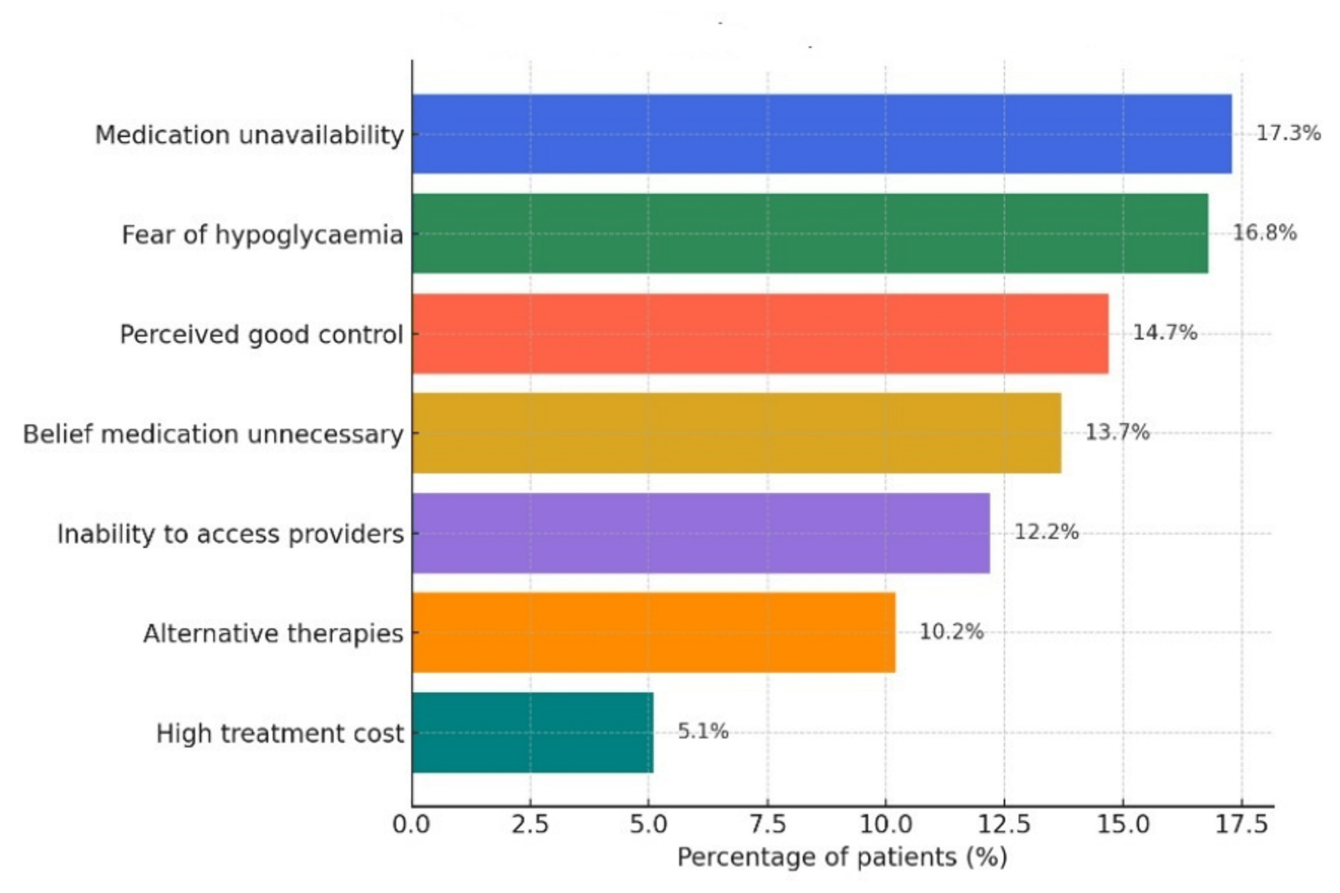
Reasons for medication discontinuation

Among the 197 respondents who provided detailed information on reasons for discontinuation (Figure 3), the most frequently reported reasons were medication unavailability (34/197; 17.3%), fear of hypoglycemia (33/197; 16.8%), perceived good glycemic control (29/197; 14.7%), and belief that medication was unnecessary (27/197; 13.7%). Additional reasons included inability to access a healthcare provider (24/197; 12.2%), initiation of alternative therapies (20/197; 10.2%), and high treatment cost (10/197; 5.1%).

Patient Attitudes and Awareness

**Figure 4:**
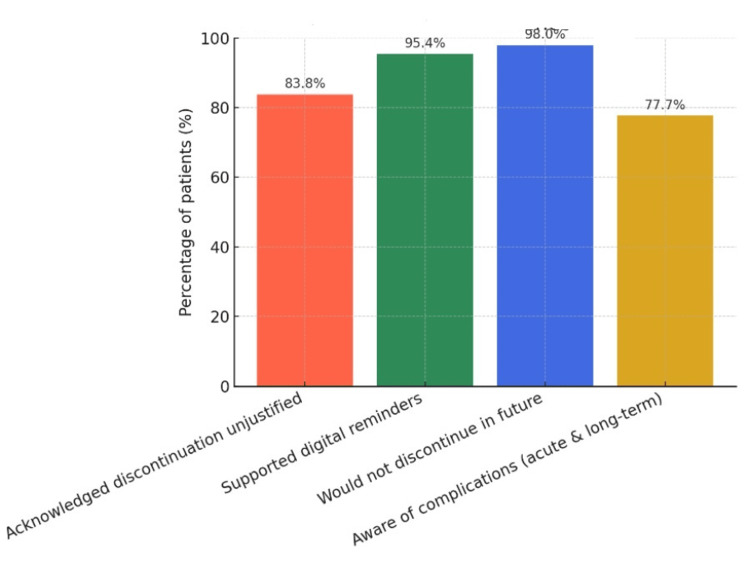
Patient awareness in the study population

Most respondents acknowledged the unjustified nature of discontinuation (83.8%, 165/197). Nearly all participants (95.4%, 188/197) supported the use of digital reminders, and 98% (199/203) stated they would not discontinue medications in the future (Figure 4). Awareness of risks was high: 77.7% (153/197) recognized that discontinuation and uncontrolled glucose levels were associated with both acute complications (e.g., infections, altered consciousness) and long-term complications (e.g., blindness, renal failure, neuropathy, cardiovascular disease).

## Discussion

This multicentric study represents one of the first systematic efforts in India to define, measure, and analyze treatment discontinuation among individuals with type 2 diabetes mellitus (T2DM) using a psychometrically validated tool. By operationalizing discontinuation as the cessation of all prescribed anti-diabetes medications for more than seven consecutive days and by developing and validating the DDD questionnaire, our study addresses a phenomenon distinct from general medication non-adherence.

Principal findings

One in four patients (25.3%) reported at least one discontinuation episode, with nearly half of these lasting one month or longer and almost 30% exceeding one year. These prolonged interruptions represent a clinically distinct phenomenon from occasional missed doses and carry greater metabolic consequences. Drivers were predominantly modifiable factors, knowledge gaps, access barriers, and behavioral patterns, rather than socioeconomic constraints.

A particularly noteworthy finding was that patients with better current glycemic control (lower HbA1c, fasting, and post-prandial glucose) were more likely to discontinue therapy. This aligns with the “perceived wellness paradox,” where symptomatic improvement leads patients to believe treatment is unnecessary. This highlights the need for sustained patient education on diabetes as a lifelong condition requiring uninterrupted therapy regardless of current control.

While age, behavioral risk factors, and microvascular complications showed statistically significant correlations with discontinuation, the strength of these associations was weak. This suggests that discontinuation behavior is unlikely to be driven by any single factor and is instead shaped by the cumulative interaction of clinical, behavioral, and contextual influences.

Comparison with global discontinuation patterns

Our discontinuation prevalence (25.3%) is consistent with international reports. The DISCOVER study across 37 countries found metformin discontinuation rates of 15.1%, with regional variation from 6.9% in Africa to 20.6% in South-East Asia [17]. A US cohort of 82,000 patients reported that 38% discontinued second-line diabetes medications within one year, rising to 50% for GLP-1 receptor agonists [18]. In Sweden, 27.9% of patients on newer agents discontinued within one year, increasing to 45.9% at three years [19].

Regional context and contributing factors

Most Indian and regional studies report non-adherence rather than complete discontinuation, with adherence rates ranging from 38% to 93% depending on the tool used [20,21]. By focusing on sustained cessation, our study provides a more precise view of treatment interruption. The predominance of access-related factors in our sample, medication unavailability (17.3%), and inability to access providers (12.2%) contrasts with high-income settings where cost-related non-adherence affects up to 16-20% of US adults with diabetes [4]. This reflects contextual differences: while cost was minor in our study (5.1%), supply-chain disruptions and healthcare access barriers are well-documented challenges in India and other low- and middle-income countries.

Our results resonate with qualitative research from Japan, which identified four discontinuation patterns: economic rationality, proactive information seeking, health professional-patient relationships, and sustained partnerships with community providers [22]. The overlap reinforces that treatment interruption is a multidimensional behavior influenced by systems, beliefs, and relationships.

The primary contribution of our study lies not in documenting discontinuation prevalence alone, but in introducing a validated, context-appropriate instrument to systematically measure this behavior in routine diabetes care in India. Unlike existing adherence tools, which primarily assess missed doses or general self-care behaviors, the DDD questionnaire captures sustained treatment cessation, its duration, and patient-reported reasons. This distinction enables clearer differentiation between transient non-adherence and clinically meaningful discontinuation, a gap that has limited prior research and intervention design in the Indian setting.

Protective role of cardiovascular medications

The protective effect of concurrent antihypertensives, statins, and aspirin suggests that integrated cardiometabolic care fosters better persistence. Similar findings have been reported in multimorbidity research, where patients engaged in comprehensive management plans show higher adherence across therapeutic domains. Rather than regimen complexity reducing adherence, our findings suggest that structured, multi-drug management under consistent supervision may reinforce continuity.

Digital health solutions and implementation opportunities

Nearly all patients (94%) endorsed digital reminder systems, highlighting an actionable opportunity. Meta-analyses demonstrate that SMS reminders improve adherence in T2DM, and randomized trials in India have shown feasibility and positive effects on adherence behaviors. With widespread mobile penetration, text messaging or app-based solutions could be rapidly implemented at scale to address knowledge and access barriers [23].

Clinical and policy implications

The high prevalence of prolonged discontinuation underscores the need to incorporate systematic screening for treatment gaps into routine diabetes care. A brief question on discontinuation history, alongside HbA1c review, could identify high-risk patients. Policy efforts should prioritize the following: (1) education on the chronic nature of diabetes and risks of discontinuation; (2) supply chain strengthening to minimize stock-outs of essential medicines; (3) digital health adoption as low-cost, scalable adherence support; and (4) integrated care models that bundle diabetes management with cardiovascular risk reduction.

These findings align with cascade-of-care analyses in South Asia showing that 64-95% of patients with diabetes have unmet care needs [24]. By characterizing discontinuation as a distinct contributor, our study provides specific intervention targets within this broader care gap.

Methodological strengths and limitations

This study has several limitations that should be considered when interpreting the findings. First, discontinuation history was assessed using self-report, which may be subject to recall bias or social desirability bias. Participants may have underreported discontinuation episodes, particularly prolonged interruptions, potentially leading to conservative estimates of prevalence. Second, the cross-sectional design limits causal inference and precludes assessment of temporal relationships between identified factors and discontinuation behavior. As a result, observed associations should be interpreted as correlational rather than predictive. Third, because discontinuation status and clinical parameters were measured at a single time point, glycemic differences between groups may reflect both prior discontinuation and concurrent disease severity, potentially attenuating or inflating observed effect sizes. Finally, although the questionnaire was rigorously validated, it was administered in Hindi and English only, which may limit generalizability to populations speaking other Indian languages.

Despite these limitations, the study provides a pragmatic and scalable approach to measuring medication discontinuation and offers clinically relevant insights into an under-recognized behavior in diabetes care.

Future directions

Longitudinal studies are needed to link discontinuation episodes with clinical outcomes, complications, and healthcare utilization. Pragmatic trials should evaluate digital reminders, structured education, and integrated care models for their impact on reducing discontinuation. Cross-cultural adaptation of the DDD questionnaire to other South Asian and global contexts would expand its utility. Predictive models using identified risk factors (younger age, better current control, behavioral factors) could enable proactive identification of high-risk patients, and cost-effectiveness analyses of interventions would inform scalable policy adoption.

Validation of the questionnaire in other Indian languages and international populations would enhance its research and clinical utility. Integration with electronic health records and telemedicine platforms could facilitate routine discontinuation monitoring.

## Conclusions

Anti-diabetes medication discontinuation represents a prevalent and often prolonged phenomenon affecting one in four diabetes patients in this Indian cohort. Knowledge deficits, access barriers, and behavioral factors, rather than socioeconomic constraints, constitute primary drivers. The validated DDD questionnaire provides a reliable assessment tool enabling systematic evaluation and targeted interventions.

Patient enthusiasm for digital reminder systems, combined with high awareness of complication risks, suggests that targeted educational interventions supported by technology-enabled adherence tools could significantly reduce discontinuation rates. Healthcare systems should prioritize routine discontinuation assessment, patient education emphasizing lifelong treatment necessity, and implementation of digital adherence support systems. These findings underscore that diabetes medication discontinuation is a complex behavioral phenomenon requiring multifaceted interventions addressing knowledge gaps, access barriers, and individual risk factors rather than solely focusing on economic constraints.

## Appendices

Diabetes Drug Discontinuation Questionnaire

This questionnaire refers to any previous discontinuation of diabetes drug therapy for more than 7 days since you have started them. Discontinuation for less than 7 days due to any reason like non-availability of drugs, forgetting few doses, outstation visits or any other short-term reason are not to be considered as discontinuation of treatment. You have to answer all the questions. For each question, tick only the most appropriate answer as per your opinion. You have to answer regarding your allopathic drug therapy only and do not consider any alternative drug therapy like home remedies, ayurvedic or homeopathic treatment Please fill the questionnaire only if you ever stopped your antidiabetic medicines for more than 7 days since you have started them, otherwise tell the average number of days you miss your doses.

1. How many times have you stopped your diabetes medicines since you first started them?

i. 1

ii. 2

iii. 3

iv. 4 or more times

2. Duration of the first discontinuation of all medicines

i. More than 7 days and extending up to one month

ii. More than 1 month and extending up to 6 months

iii. More than 6 months and extending up to 1year

iv. More than one year

3. Before the first discontinuation, how long did you take treatment as advised by your doctor?

i. Less than 1 week

ii. One week to one month

iii. One month to one year

iv. More than one year

4. In your view, what was your level of sugar control when you stopped your antidiabetic drugs?

i. Good

ii. Not bad

iii. Very bad

iv. Sugar levels went low

5. On whose advice did you first start allopathic medicine?

i. Qualified allopathic doctor

ii. Unqualified practitioner/ pharmacist

iii. Family member or friend

iv. Electronic media or internet search

6. Why did you stop drug therapy?

i. On advice as stated in the above question number 5

ii. High cost of treatment (Inability to afford medicines) or

iii. Short supply of medicines

iv. Unable to reach the doctor

v. Greater number of pills

vi. Low sugar levels

vii. GI upset or any other event

viii. The doctor advised starting insulin

ix. Started alternate treatment

x. Thought that it is not necessary to take medicines on a regular basis

xi. Prolonged religious fasting

xii. Afraid of the side effects of the medicine

xiii. Good control of sugar level was achieved

xiv. Opposed by the family members

xv. Depression due to a longer duration of treatment

7. What happened after stopping treatment

i. Sugar level remained well-controlled

ii. Sugar levels increased

iii. Complications occurred

iv. Don't know

8. Besides discontinuation of medicines, which of the following do you do without a doctor’s advice?

i. Reduce the dose of medicines

ii. Discontinue some of the drugs

iii. Start taking some other medicine

iv. None of the above

9. Do you know that discontinuation of treatment and uncontrolled sugar are associated with?

i. Acute complications like infections, poor physical and mental abilities, and altered consciousness

ii. Long-term complications like blindness, kidney failure, nerve diseases, and heart diseases

iii. Associated with both of the above

iv. Not associated with any of the above

10. When you last discontinued your treatment for more than 7 days

i. Still off medicines

ii. Discontinued in the last month but started again now

iii. Discontinued in the last 6 months but started again now

iv. Discontinued in the last 1 year but started again now

v. Discontinued more than 1 year back, but started again now

11. Purpose of the present visit is

i. Uncontrolled diabetes or side effects

ii. Want to know the current status of diabetes and need to start treatment if needed

iii. I am now taking anti-diabetes medicines regularly and this is a follow-up visit

iv. I want a consultation for other problems

12. Are you convinced that your previous treatment discontinuation was a justified step?

Yes/No

13. Do you think that digital reminders (such as email, SMS, or WhatsApp) could help you improve compliance?

Yes/No

14. Will you ever take a chance to stop all antidiabetic medicines in the future?

Yes/No

Please write below anything important you want to tell your doctor: ________________________________________________________________________ ________________________________________________________________________
